# Innate Immune System and Multiple Sclerosis. Granulocyte Numbers Are Reduced in Patients Affected by Relapsing-Remitting Multiple Sclerosis during the Remission Phase

**DOI:** 10.3390/jcm9051468

**Published:** 2020-05-14

**Authors:** Zbyšek Pavelek, Francesco Angelucci, Ondřej Souček, Jan Krejsek, Lukáš Sobíšek, Blanka Klímová, Jana Šarláková, Simona Halúsková, Kamil Kuča, Martin Vališ

**Affiliations:** 1 Department of Neurology, Faculty of Medicine and University Hospital Hradec Králové, Charles University in Prague, Sokolská 581, 500 05 Hradec Kralove, Czech Republic; fangelucci@hotmail.com (F.A.); lukas.sobisek@yahoo.com (L.S.); blanka.klimova@uhk.cz (B.K.); jana.sarlakova@fnhk.cz (J.Š.); simona.haluskova@fnhk.cz (S.H.); valismar@seznam.cz (M.V.); 2 Memory Clinic, Department of Neurology, 2nd Faculty of Medicine, Charles University and Motol University Hospital, V Úvalu 84, 150 06 Prague, Czech Republic; 3 Department of Clinical Immunology and Allergology, University Hospital Hradec Králové, Sokolská 581, 500 05 Hradec Kralove, Czech Republic; ondrej.soucek@fnhk.cz (O.S.); jan.krejsek@fnhk.cz (J.K.); 4 Department of Chemistry, Faculty of Science, University of Hradec Králové, Rokitanského 62, 500 03 Hradec Kralove, Czech Republic; kamil.kuca@uhk.cz; 5 Biomedical Research Center, University Hospital Hradec Kralove, Sokolská 581, 500 05 Hradec Kralove, Czech Republic

**Keywords:** multiple sclerosis, relapsing-remitting, granulocytes, monocytes, CD64, PD-L1

## Abstract

Background: Multiple sclerosis (MS) is a neurodegenerative disease that affects the central nervous system. The cause of MS is still unknown, and the role of innate immunity is still poorly understood. Objective: The goal of this study was to understand whether, compared to healthy controls, the elements of innate immunity are altered in the blood of MS patients in the remitting phase. Methods: A total of 77 naïve MS patients and 50 healthy controls were included in this cohort study. Peripheral blood samples were collected and analyzed. All the calculations were performed with the statistical system R (r-project.org). Results: The results showed that MS patients had significantly lower relative representations of granulocytes than healthy controls, while the relative representations of monocytes remained unchanged. CD64- and PD-L1-positive granulocytes exhibited a nonsignificant decreasing trend, while granulocytes with other membrane markers remained noticeably unchanged. Conclusion: The results of this study suggest that studies of the causes of MS and its treatment should also be focused on the elements of the innate immune response.

## 1. Introduction

Multiple sclerosis (MS) is a neurodegenerative disease that affects the central nervous system (CNS). MS is characterized by anomalous immune responses that attack some components of the CNS, mistaking them for foreign agents [1]. The inflammatory process triggered by the immune system can damage the myelin sheath that surrounds and isolates nerve fibers and the cells specialized in its production (oligodendrocytes) [2]. This process, called demyelination, can cause areas of myelin loss or injury, which are called plaques, particularly in the optic nerves, cerebellum and spinal cord [3].

The demyelination process causes several different neurological symptoms, depending on the brain area affected [1]. The most common symptoms are autonomic, visual, motor, and sensory problems [1]. According to the symptomology, MS is classified into three main types: relapsing-remitting, primary progressive and secondary progressive [4]. Relapsing-remitting MS is characterized by two phases. In the relapse phase, there are distinct attacks of neurological symptoms which can last from few days to some months [4]. During the remission phase, the symptoms fade away either partially or completely. Around 85% of people with MS are diagnosed with this type. Relapsing-remitting MS is usually followed by secondary progressive MS, which is characterized by a steady state of disability, independent of any relapses [5]. Primary progressive MS affects about 10–15% of people diagnosed with MS. People with primary progressive MS can experience many symptoms of relapsing-remitting MS, but symptoms gradually get worse over time, rather than appearing as sudden relapses [2]. 

MS is considered a multifactorial disease, as different factors can be involved in its onset [6]. Some studies suggest that factors related to the environment, ethnicity, genetic predisposition and infectious agents might be involved in MS onset [7,8]. The pathological mechanism causing MS symptoms is still under investigation. It is known that when myelin is engulfed by macrophages, the axons of the nerves remain bare, and they are no longer able to efficiently transmit electrical impulses. Many alterations affecting different types of cells of the immune system, such as T cells and B cells, have been reported [9]. However, the relationship between neuroinflammation and neurodegeneration and their contribution to the different stages of the disease are still unclear [10].

Acquired immunity (mediated by lymphocytes) plays a clearly recognized role in the pathology of MS [6]. However, in recent years, the role of innate immunity in MS pathology has been increasingly recognized [11]. In addition, recent studies have identified over two hundred regions of the human genome that influence a large number of different immune cells, highlighting the fact that this disease is not caused by a single immune cell type but rather by extensive immune system dysfunction [12,13].

Innate immunity starts inflammatory processes and is composed of a multitude of biochemical and cellular defense mechanisms ready to react after an initial stimulus (for example, infectious or traumatic stimuli) [14]. Important cells of the innate immune system are mononuclear phagocytes (monocytes, macrophages and dendritic cells), granulocytes (neutrophil, basophil and eosinophil), mast cells and natural killer (NK) cells [15]. These cells may have a relevant role in the initiation and progression of MS [16,17]. Mononuclear phagocytes, such as microglia and macrophages, are the dominant immune cells located in MS lesions in both relapsing-remitting and progressive phases of the disease [16,17]. These cells interact with cells of the adaptive immune system (such as T cells and B cells), but can also directly cause neuroinflammatory tissue damage through several mechanisms, including secretion of proinflammatory cytokines, chemokines, free radicals, and increased release of glutamate [18]. In addition, activated microglia and macrophages in active MS lesions induce oxidative stress [19] and axonal degeneration [20] that contribute to both grey and white matter pathology [21]. According to these findings, it has been proposed that many of these innate cell types may represent a therapeutic target for MS [22]. However, although phagocytes can drive tissue destruction during lesion formation, studies using animal models of CNS injury suggested that these cells might also be a necessary part of the tissue repair mechanism during lesion resolution [18]. In murine MS models, it has been evidenced that monocyte-derived phagocytes seem to initiate demyelination, whereas microglia-derived phagocytes clear cellular debris and thus promote tissue recovery [23].

Mononuclear phagocytes are not the only type of innate immune cells investigated in MS. The involvement of granulocytes in MS has also emerged in the last few years. Granulocytes are white blood cells that help the immune system fight off infection [24]. Typically, granulocytes play a role both in innate and adaptive immune responses in the fight against viral and parasitic infections [25]. As part of the immune response, granulocytes migrate to the site of infection and release a number of different effector molecules, including histamine, cytokines, chemokines, enzymes and growth factors. As a result, granulocytes are an integral part of inflammation and play a significant role in the etiology of allergies [26]. However, the specific role of neutrophils in MS is still not well defined. In addition, despite the massive amount of data on the inflammatory phase of MS, little is known about the levels of innate cells in non-active MS phases, such as the remission phase. 

Thus, in this study, we investigated by flow cytometry whether the number of granulocytes and monocytes in MS patients in the remitting phase is different from that of healthy controls. Different flow cytometric biomarkers were used to better characterize these immune cell populations. 

## 2. Material and Methods

### 2.1. Study Population

The participating subjects were all patients of the Department of Neurology of the University Hospital in Hradec Králové, Czech Republic. The study was conducted from January 2018–December 2019. Seventy-seven Caucasian patients with relapsing-remitting MS (or after the first demyelinating event) and 50 healthy controls (HC) without any comorbidities were included in this cohort study. All the included MS patients met the McDonald criteria or the revised McDonald criteria for MS and were in the remission phase (baseline was at least 30 days after relapse) [27]. MS patients had not yet been treated with disease-modifying drugs (DMDs) such as interferons, teriflunomide or glatiramer acetate. Although DMDs are not a cure for MS, they can reduce the number of relapses and slow down the damage caused by relapsing MS that builds up over time [28].

All the participants provided written informed consent. The study protocol was approved by the Ethical Committee of the University Hospital Hradec Králové, reference number 201801S08P.

### 2.2. Data Collection and Analysis

**Figure 1:**
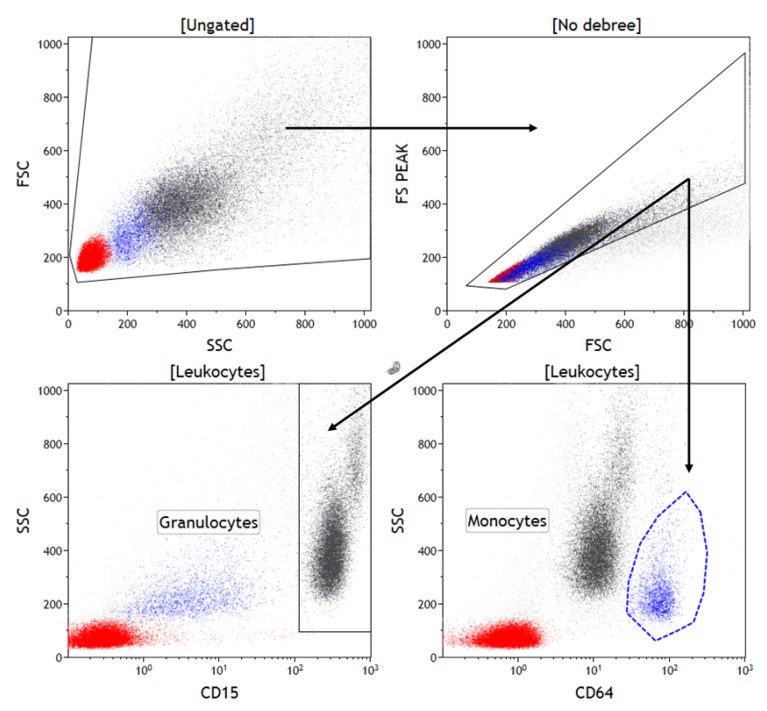
Gating strategy for granulocytes and monocytes **Legend**. Gating strategy: Obtained events were gated in an FSC intensity and SSC intensity dot plot to eliminate debris. Cells were gated on an FSC intensity and FSC peak dot plot to eliminate doublets. Granulocytes were gated on an SSC vs. CD15 dot plot. Monocytes were gated on an SSC vs. CD64 dot plot. The expression level of selected markers is evaluated as fluorescence intensity on the histograms. FSC: Forward scatter, SSC: Side scatter.

The authors collected blood samples from the antecubital fossa vein. For the surface staining of leukocytes, 50 µL of anticoagulated blood was added to tubes containing 5 µL of fluorochrome-labeled monoclonal antibodies, including anti-CD11b allophycocyanin (APC), clone Bear1; anti-CD14-APC, clone M5E2; anti-CD15 fluorescein isothiocyanate (FITC), clone 80H5; anti-CD16 R-phycoerythrin (RPE), clone 3G8; anti-CD62L-RPE, clone DREG56; anti-CD64-RPE, clone 22; anti-CD163-FITC, clone GHI/61; and anti-CD274-APC, clone PD-L1. The anti-CD163-FITC and anti-CD14-APC were manufactured by BD Biosciences (Franklin Lakes, NJ, USA), and all the other antibodies were manufactured by Beckman Coulter (Miami, FL, USA). The blood samples were incubated with the antibodies for 15 min at room temperature in the dark. Then, a lysing solution (OptiLyse C, Beckman Coulter, Miami, FL, USA) was added, and the mixture was incubated for another 10 min. The flow cytometric evaluation was conducted with a Navios 10 flow cytometer (Beckman Coulter, Miami, FL, USA). The gating strategy for the granulocytes and monocytes is presented in Figure 1.

### 2.3. Statistical Analysis

All the data were then assessed using the Kaluza C 1.1 analysis software (Beckman Coulter). Data on a minimum of 50,000 events were obtained for each staining sample and were supplied as a list mode. The gating strategies for the different leukocyte subsets that were assessed were as follows: lymphocytes (low SSC/CD45++), granulocytes (CD45+ and CD15+ or CD16+), and monocytes (CD45+ and CD14+ or CD64+). The intensity of the expression of CD11b, CD14, CD15, CD16, CD64, CD163, CD62L and PD-L1 was evaluated as the mean fluorescence intensity (MFI).

The observed characteristics (parameters) were detected/measured spatially (cross-sectional, i.e., at one time point) for 77 patients with MS (RS) and 50 healthy controls (HC). The empirical distribution of two (potential) covariates differed between these groups: sex (HC: 74.0% women; MS: 63.6% women) and age (HC average: 43.7 years; MS average: 35.7 years). To reduce the risk of impact of these covariates on the endpoints, the RS patients were paired with HC according to these two covariates by the propensity score matching method, which was implemented in the MatchIt package [29]. Then, the parameters were compared between a paired group of 50 RS patients and a group of 50 HC using statistical tests. A parametric *t*-test was used to compare the group means for the normally distributed characteristics. The effect size (standardized difference) was quantified using Cohen’s D. The bilateral alternative hypothesis and the 5% significance level were chosen for the statistical tests. All the calculations were performed in the R statistical system version 3.5.2 [30].

## 3. Results

### 3.1. Demographic Characteristics

There were no significance differences in sex ratio (males/females) between MS patients and healthy controls. The MS group was significantly younger (mean age 38.4) than the HC group (mean age 38.4) (Table 1).

### 3.2. Flow Cytometric Evaluation of Immune Cell Biomarkers

There was a significant difference for the parameter granulocytes—relative representation because MS group had lower values as compared to HC group [MS: mean (SD) 52.8 (10.7); HC: 57.2 (10.4); *p* value = 0.041; size effect = 0.21]. 

In the MS group, there was a non-significant trend to decrease for the following three biomarkers: CD64 granulocytes [MS: mean (SD) 4.1 (1.9); HC: 4.9 (3.1); *p* value = 0.130; size effect = 0.15], PD-L1 granulocytes [MS: mean (SD) 0.84 (0 (23); HC: 1.02 (0.71); *p* value = 0.173; size effect = 0.17], and lymphocytes—relative representation (MS: mean (SD) 36.3 (10.3); HC: 32.7 (9.6); *p* value = 0.072; size effect = 0.18). The results relative to these and the other biomarkers utilized are reported in Table 1.

## 4. Discussion

This study was performed to investigate whether cells of the innate immune response are altered in MS patients in remission phase as compared to healthy subjects. We used flow cytometry to analyze granulocytes and monocytes in MS patients and controls in order in to determine their relative representation and to distinguish their subtypes with various markers. The results showed that MS patients had significantly lower relative representations of granulocytes than healthy controls. By using the markers, it was observed that the CD64- and PD1L-positive granulocytes exhibited a nonsignificant decreasing trend. The relative representation of monocytes did not differ from controls, while lymphocytes were increased in MS patients, but not significantly. 

Our data demonstrate that the number of granulocytes is reduced during remission. There are four types of granulocytes: basophils, eosinophils, neutrophils and mast cells [31]. The use of biomarkers suggests that, probably, the type of granulocyte reduced in our MS patients is the neutrophils. This idea is supported by the fact that CD64 is expressed in neutrophil granulocytes and is upregulated during inflammatory processes and septic complications [32]. CD64, also called FcγRI (Fc γ receptor I), is a class of plasma membrane receptors expressed on human myeloid cells [33]. CD64 contains three extracellular immunoglobulin-like domains that represent binding sites for the Fc portion of IgG. The FcγRI receptor is essential for at least the start of the phagocytosis. Phagocytosis is a multistep process, several orders more effective in the presence of so-called opsonins. Opsonins are specific IgG antibodies bound to the surface of engulfed particles, making the bridge via interaction with FcγRI receptors expressed on the surface of granulocytes. It has been recently observed that the presence of IgM antibodies against CD64 in the blood of MS patients seems to be associated with a significantly lower annualized relapse rate and with the improved maintenance of clinical stability compared to patients without these antibodies [34]. Natural antibodies reacting with CD64 are presumably blocking these high-affinity receptors for IgG with numerous consequent impacts on inflammatory response. 

Recent evidence indicates that programmed death ligand 1 (PD-L1) [35] is also expressed on neutrophils and is associated with the development of numerous diseases, including autoimmune diseases such as systemic lupus erythematosus [36]. In EAE models, it has been shown that amelioration induced by epigenetic drugs is associated with a reduction in PD-L1-positive neutrophils during the preclinical phase [37].

Thus, the data on these biomarkers confirm that the subpopulation of granulocytes undergoing a nonsignificant reduction are presumably immune-activated neutrophils. Neutrophils have been extensively studied in the general context of neuroinflammation. For example, in amyotrophic lateral sclerosis, their numbers are elevated and correlate with disease progression [38]. It is known that, during disorders such as MS and Alzheimer’s disease, neutrophils can migrate to the CNS, acquire a toxic phenotype, home in on neurons, and release harmful molecules that compromise neuronal functions [39,40]. However, the specific role of neutrophils in MS is still not well defined. In rodent models of MS, it has been shown that neutrophils can favor the onset and increase the severity of experimental autoimmune encephalomyelitis (EAE) [41,42]. In this model, when neutrophils are depleted, or the actions of their mediators are blocked, the severity of EAE is reduced, indicating that these cells may play an important role in the pathogenesis of MS. It has been also demonstrated that one of the potential methods by which neutrophils contribute to EAE pathology is by facilitating the breakdown of the blood–brain barrier (BBB), as depletion of neutrophils restores BBB integrity [43]. 

In MS patients, the levels of neutrophils have been measured in the blood and cerebrospinal fluid (CSF). In the CSF, increased neutrophil levels have been found in MS patients during relapse [44]. Interestingly, neutrophil levels are higher in pediatric patients, while in adults, neutrophil levels decrease with disease duration, suggesting activation of the innate immune system in early disease [44,45]. The levels of neutrophils in the blood are also altered in MS. It has been shown that the neutrophil-to-lymphocyte ratio increases during the relapse phase of MS, and this ratio has been proposed as a marker of MS disease activity [46,47]. Upregulated expression of PD-L1 was observed in the lesions of brain specimens from MS patients, demonstrating the critical importance of B7-H1 as an immune-inhibitory molecule that is capable of downregulating T cell responses [48]. 

These data suggest that neutrophils are increased during the inflammatory phase of MS and contribute to sustaining the inflammatory process by releasing inflammatory mediators [42]. In remission phase, we observe a significant reduction of neutrophils in the blood of our MS cohort. These findings are in line with data showing that peripheral blood mononuclear cells from relapsing-remitting MS patients in remission phase exhibit decreased PD-L1 expression [49]. Our interpretation of (non-significantly) decreased expression of PD-L1 on granulocytes of our MS patients in remission reflects the current paradigm of MS as Th1 and Th17 pathology. A subset of Th1 T cells is the principal source of interferon γ, which is a potent stimulator of PD-L1 expression on immune cells. As clinical remission is achieved in our MS patients by immunomodulatory therapy, it seems likely that interferon γ production is diminished, with a subsequent decrease in the PD-L1 presence on granulocytes. 

There are some limitations to the interpretation of our data. First, these data represent the totality of granulocytes (not only neutrophils). Although CD64- and PD1L are markers of neutrophils, they can also bind to the membranes of other cell types of granulocytes. Another limitation is that we did not include the patients in relapse phase. The third limitation is the low number of patients included. Thus, our data should be regarded as preliminary findings and need to be confirmed in a larger cohort, possibly including the same patients in relapse phase, and using other specific biomarkers.

## 5. Conclusions

This study shows that neutrophils are decreased in MS patients in the remission phase. Despite the mounting evidence for a role of neutrophils in MS, it is not clear whether they contribute to disease initiation, pathogenesis, and/or relapse. Nonetheless, these findings suggest that decreasing neutrophil activity during the early onset of MS could represent a potential therapeutic strategy. 

## Figures and Tables

**Table 1:** Descriptive statistics for matched multiple sclerosis (MS) patients and healthy controls (HC).

Parameter	Experimental Groups						Statistics		
	HC			MS					
	*mean*	*SD*	*median*	*mean*	*SD*	*median*	*test*	*p Value*	*Size Effect*
**Age**	**43.74**	11.89	46.5	**38.36**	9.78	38	*	0.01528	0.25
**Granulocytes**									
Relative representation	**57.2**	10.44	58.41	**52.82**	10.66	53.16	*	**0.0412**	0.21
CD11b	**84.28**	15.33	83.33	**87.98**	21.99	86.22	*	0.3346	0.1
CD64	**4.92**	3.07	5.58	**4.14**	1.85	4.16	*	0.1302	0.15
CD62L	**34.52**	9.9	35.63	**36.26**	10.37	35.78	*	0.3946	0.09
PD-L1	**1.02**	0.71	0.85	**0.84**	0.23	0.82	**	0.1733	0.17
CD14	**1.78**	0.4	1.71	**1.88**	0.71	1.71	**	0.8501	0.08
CD16	**626.75**	120.41	620.35	**666.74**	163.15	675.92	*	0.168	0.14
**Monocytes**									
Relative representation	**5.83**	1.73	5.76	**5.28**	2.28	4.49	*	0.1798	0.14
CD11b	**79.65**	20.71	73.74	**81.88**	20.86	78.85	**	0.4393	0.05
CD15	**4.83**	2.88	4.03	**5.99**	4.59	3.99	**	0.4089	0.15
CD62L	**25.43**	7.35	25.28	**27.06**	8.54	25.31	*	0.3131	0.1
PD-L1	**0.99**	0.82	0.73	**0.87**	0.4	0.74	**	0.7688	0.09
CD163	**3.44**	0.77	3.45	**3.7**	1.16	3.49	**	0.427	0.14
CD16	**5.74**	6.12	3	**8.32**	12.2	2.41	**	0.9581	0.13
**Lymphocytes**									
Relative representation	**32.67**	9.63	32.95	**36.32**	10.34	35.28	*	0.07218	0.18

Legend: The following statistical tests were conducted to compare differences between groups: (*) parametric *t*-test; (**) nonparametric *t*-test. Effect size was assessed by Cohen’s D.
